# Effects of Maternal Inflammation and Exposure to Cigarette Smoke on Birth Weight and Delivery of Preterm Babies in a Cohort of Indigenous Australian Women

**DOI:** 10.3389/fimmu.2015.00089

**Published:** 2015-03-10

**Authors:** Kirsty G. Pringle, Kym Rae, Loretta Weatherall, Sharron Hall, Christine Burns, Roger Smith, Eugenie R. Lumbers, C. Caroline Blackwell

**Affiliations:** ^1^School of Biomedical Sciences and Pharmacy, Faculty of Health and Medicine, University of Newcastle, Newcastle, NSW, Australia; ^2^Hunter Medical Research Institute, Newcastle, NSW, Australia; ^3^Department of Rural Health, Faculty of Public Health and Medicine, University of Newcastle, Newcastle, NSW, Australia; ^4^Gomeroi gaaynggal Centre, Tamworth, NSW, Australia; ^5^Mothers and Babies Research Centre, Faculty of Public Health and Medicine, University of Newcastle, Newcastle, NSW, Australia; ^6^Information-Based Medicine, School of Biomedical Sciences and Pharmacy, Faculty of Health and Medicine, University of Newcastle, Newcastle, NSW, Australia; ^7^Hunter Area Pathology Service Immunology, Newcastle, NSW, Australia

**Keywords:** Indigenous, pregnancy, SIDS, inflammation, smoking

## Abstract

Sudden infant death syndrome (SIDS), neonatal deaths, and deaths from infection are higher among Indigenous Australians. This study aimed to determine the effects of inflammatory responses and exposure to cigarette smoke, two important factors associated with sudden death in infancy, on preterm birth, and birth weight in a cohort of Indigenous mothers. Indigenous Australian women (*n* = 131) were recruited as part of a longitudinal study while attending antenatal care clinics during pregnancy; blood samples were collected up to three times in pregnancy. Serum cotinine, indicating exposure to cigarette smoke, was detected in 50.4% of mothers. Compared with non-Indigenous women, the cohort had 10 times the prevalence of antibodies to *Helicobacter pylori* (33 vs. 3%). Levels of immunoglobulin G, antibodies to *H. pylori*, and C-reactive protein (CRP) were all inversely correlated with gestational age (*P* < 0.05). CRP levels were positively associated with maternal body mass index (BMI; ρ = 0.449, *P* = 0.001). The effects of cigarette smoke (cotinine) and inflammation (CRP) were assessed in relation to risk factors for SIDS: gestational age at delivery and birth weight. Serum cotinine levels were negatively associated with birth weight (ρ = −0.37, *P* < 0.001), this correlation held true for both male (ρ = −0.39, *P* = 0.002) and female (ρ = −0.30, *P* = 0.017) infants. Cotinine was negatively associated with gestational age at delivery (ρ = −0.199, *P* = 0.023). When assessed by fetal sex, this was significant only for males (ρ = −0.327, *P* = 0.011). CRP was negatively associated with gestational age at delivery for female infants (ρ = −0.46, *P* < 0.001). In contrast, maternal BMI was significantly correlated with birth weight. These data highlight the importance of putting programs in place to reduce cigarette smoke exposure in pregnancy and to treat women with chronic infections such as *H. pylori* to improve pregnancy outcomes and decrease risk factors for sudden death in infancy.

## Introduction

Among Indigenous Australians, there is a greater risk of sudden infant death syndrome (SIDS) compared with non-Indigenous Australians (1). This is perhaps not surprising as low birth weight and preterm birth, both risk factors for SIDS, are approximately twice as common among Indigenous Australians compared with non-Indigenous families (2). Additional perinatal risk factors for SIDS include poverty and exposure to cigarette smoke (3, 4), factors also more common among Indigenous Australians.

The burden of infection within the Indigenous population is high and accounts for 5% of the total burden of disease (5). Deaths due to infection are significantly more common among Indigenous Australian adults, particularly bacterial infections (6). Mild infection, either from a “cold,” or an infection of a gastric nature, is often reported by parents of SIDS infants; and the risk factors for SIDS parallel those for infection (see Blackwell et al., this issue).

Infection stimulates the inflammatory response. The cytokines in turn stimulate the acute phase response and the endocrine production of the powerful anti-inflammatory hormone, cortisol. We have previously demonstrated that a central Australian Indigenous community had single nucleotide polymorphisms (SNPs) for cytokine genes that differed significantly from those of non-Indigenous Australians, and this SNP profile might contribute to powerful pro-inflammatory responses implicated in host responses to infection (7). *In vitro* studies using peripheral blood mononuclear cells indicated that inflammatory responses elicited in response to the bacterial antigen lipopolysaccharide (LPS) were significantly affected by the sex of the donor, cytokine SNP profile, and surrogates for viral infection and cigarette smoke (8, 9).

Evidence of strong inflammatory responses among Indigenous Australians has also been reported. C-reactive protein (CRP) is an acute phase protein that is stimulated by infection and CRP levels rise during pregnancy (10). There are significant differences in levels of CRP associated with ethnic groups; black women in the United States had higher CRP levels than white women (7.68 vs. 2.59 mg/L) (11). Among Indigenous women in a remote community, median CRP levels were reported to be markedly higher (8 mg/L) (12) than those reported for American women in the Women’s Health Study (1.5 mg/L) (13) and levels significantly increased with age.

One of the major risk factors for poor pregnancy outcome and infant deaths is maternal cigarette smoking during pregnancy. Due to the extended family arrangements in which many Indigenous women live, passive exposure to cigarette smoke is an important factor to consider. Considerable work has been done to reduce smoking in Indigenous communities with little improvement in many areas. It has been reported that many Indigenous women feel that smoking will reduce their perceived stress, and there is limited understanding of the impacts of smoking on the developing child in pregnancy (14).

In this study, we examined the effects of inflammatory responses and exposure to cigarette smoke among a cohort of Indigenous mothers participating in studies of pregnancy outcome on preterm birth and low birth weight, two important factors associated with sudden death in infancy. The study has been described previously (15). Our hypothesis was that risk factors we have found *in vitro* to contribute to the dysregulation of inflammatory responses (9, 16) might be more common in the Indigenous population, and that if present, the increase in inflammation might be associated with preterm birth and/or lower birth weight. Since our previous studies indicated that testosterone levels can affect pro-inflammatory responses (9), we assessed the effect of fetal sex on maternal inflammatory markers as fetal plasma testosterone is significantly higher in males (17). To examine this hypothesis, we addressed the following questions:

1. Is there evidence for increased inflammatory responses among pregnant Indigenous women?
2. Are inflammatory markers affected by risk factors associated with poor pregnancy outcome or sudden death in infancy?
3. Does evidence of inflammation or other risk factors for SIDS affect birth weight or gestational age at birth of male and female infants?

## Materials and Methods

### Study design

This is a prospective longitudinal cohort study that was developed through a thorough community consultation and continues to be reviewed by the members of its Indigenous Steering committee. This study was approved by the Hunter New England Local Health District Human Research Ethics Committee (HNEHREC ref. no. 08/05/21/4.01, NSWHREC ref. no. 08/HNE/129), the University of Newcastle Human Research Ethics Committee (H-2009-0177), and the Aboriginal Health and Medical Research Council Ethics Committee (ref. no. 654/08).

The Indigenous women in this cohort have been recruited by Indigenous research assistants. Informed written consent was obtained in the presence of at least one family member. Recruitment of participants occurred when they were attending antenatal care during their pregnancy. The study design aimed to obtain one blood sample from each participant in each trimester, however sampling was opportunistic as there was a tendency for participants to attend clinics irregularly for care.

Some of the variables [immunoglobulin G (IgG) to *Helicobacter pylori* and cotinine] were compared with those obtained for samples from 150 healthy non-Indigenous, non-pregnant female blood donors. Approval was obtained from the Australian Red Cross Blood Sample (ARCBS) Ethics Committee (07-11NSW-07). Plasma was collected from buffy coats of 150 female blood donors for the assessment of cotinine, exposure to cigarette smoke, a confounding variable in assessment of inflammatory responses, and for evidence IgG antibodies to *H. pylori*.

### Sample collection

Venipuncture was conducted by a trained Indigenous Australian research assistant or a medical professional. Whole blood samples were analyzed immediately for white blood cell (WBC) count. Blood for biochemical analyses were collected into lithium-heparin or EDTA vacutainers as appropriate and placed on ice until serum or plasma was separated by centrifugation (3,500 rcf, 10 min) at 4°C. Samples were aliquoted and stored at −80°C until time of analysis of: CRP; total IgG, IgA, and IgM; IgG antibodies to *H. pylori*; cotinine; and cortisol.

### Surveys

Participants were surveyed on their smoking habits and those of the people in their place of residence in an effort to determine if self-reporting of exposure to tobacco is reliable.

### Biological assays

Parameters assessed in an earlier study of inflammatory responses among Indigenous Australians were used in this study (12): CRP; total immunoglobulins; WBC; IgG to *H. pylori*; exposure to cigarette smoke. In addition, the anti-inflammatory hormone cortisol was also assessed.

White blood cell count was measured from whole blood immediately following collection from participants using a Beckmann Coulter LH780 analyzer at Pathology North, Tamworth, NSW, Australia. CRP for each stored sample was determined using the Abbott Architect 8200 analyzer at Pathology North, Tamworth, NSW, Australia and total IgG, IgA, and IgM for each sample was determined by Hunter Area Pathology Service Immunology laboratory, John Hunter Hospital, New Lambton, NSW, Australia. Quantitative IgG antibodies specific for *H. pylori* were determined by adapting a commercially available quantitative enzyme linked immunosorbent assay (ELISA) kits (Bio-Rad Laboratories Inc., GAP™ IgG kit (Cat#4042002) based on previous studies relating to chronic infection and heart disease (18). Cortisol was analyzed by Pathology North, Tamworth, NSW, Australia using the Abbott Architect 4100 analyzer.

Plasma from the samples from ARCBS donors and women in the study were assessed for exposure to cigarette smoke by a semi-quantitative commercial competitive enzyme immunoassay (EIA) kit according to manufacturer’s instructions [Bio Quant Cotinine ELISA, CA. Catalog No. BQ 096D (96 wells)], as described previously (9).

### Statistics

Data are expressed as medians and ranges and were analyzed using SPSS Version 21 (Chicago). Non-parametric tests were used to determine significant differences between trimesters. Spearman’s non-parametric correlations were used to determine relationships between variables. Significance was set at 5%.

## Results

### Participant demographics

Data were obtained from 131 participants, of whom 31 had data collected at three or more visits during their pregnancies, 51 had data collected at two visits, and 49 had data collected at one visit only.

The median age of women in the Indigenous pregnancy cohort was 25 years (range of 13.8–40.9 years). The median body mass index (BMI) of the cohort was 30 (range of 15–52 kg/m^2^). The number of past pregnancies ranged from 0 to 23 and the number of live children ranged from 0 to 9; 18% had a previous miscarriage (range 2–16); 2.5% reported a past stillbirth or sudden unexpected death in infancy (SUDI).

The median gestational age at delivery was 39.1 weeks (range 32–43 weeks). The median birth weight of the infants was 3180 g (range 910–5430 g).

### Inflammation

The markers for inflammation were assessed by trimester of pregnancy (Table 1). Cortisol and cotinine were also assessed by trimester.

**Table 1:** **Markers for inflammation and smoking in each trimester of pregnancy in Indigenous Australian women**.

	Trimester			Normal range^f^
	1	2	3	
IgG (g/L)	9.69 (7.66–14.6, *n* = 9)	9.17 (6.19–17, *n* = 51)	8.43 (3.38–240, *n* = 90)^d^^,^^e^	6.45–13.9^a^
IgA (g/L)	1.67 (0.81–2.21, *n* = 9)	1.62 (0.78–2.87, *n* = 51)	1.73 (0.52–862, *n* = 90)	0.80–4.12^a^
IgM (g/L)	0.70 (0.45–1.13, *n* = 9)	1.01 (0.32–3.4, *n* = 51)^d^	1.10 (0.32–3.4, *n* = 89)^d^	0.44–2.76^a^
*H. pylori* IgG (U/mL)	11.7 (8.6–28.4, *n* = 9)	9.45 (0.56–2.8, *n* = 42)	6.4 (2–82, *n* = 43)^d^	Negative <18, equivocal 18–20, positive >20
WBC (10^9^/L)	9.9 (7–13, *n* = 13)	9.8 (3–17, *n* = 55)	10.5 (5–18, *n* = 103)	4.5–13.0^b^, 4.0–11.0^c^
CRP (mg/L)	7.3 (0.5–26.3, *n* = 11)	7.5 (0.5–109, *n* = 55)	5.3 (0.2–92, *n* = 104)^e^	<5.0
Cortisol (nmol/L)	285 (198–500, *n* = 8)	360 (151–878, *n* = 45)	441.5 (13–733, *n* = 90)^d^^,^^e^	79–535
Cotinine (ng/mL)	36.9 (0–214, *n* = 9)	40 (0–440, *n* = 49)	0.3 (0–337, *n* = 82)	N/A

*Data are presented as median (ranges, *n*)*.

*^a^>16 years*.

*^b^16–21 years*.

*^c^>21 years*.

*^d^Denotes significant difference from first trimester values*.

*^e^Denotes significant difference from second trimester values, *P* < 0.05*.

*^f^Normal non-pregnant ranges derived from Ref. (27)*.

Immunoglobulin G decreased significantly with trimester (*P* = 0.003); levels in the third trimester were significantly lower than trimester 1 or 2 (*P* = 0.012 and *P* = 0.007, respectively; Table 1). In contrast, IgM levels increased significantly with trimester (*P* = 0.025); levels in the first trimester were significantly lower than trimester 2 or 3 (*P* = 0.014 and *P* = 0.009, respectively). Levels of IgG, IgA, and IgM were higher among women carrying a female fetus, but the differences were not significant. Univariate analysis of IgG with trimester and sex as fixed factors showed that levels fell significantly with gestation and there was an interaction between sex and trimester (*P* = 0.046).

Compared with 150 non-Indigenous women of childbearing age, our cohort had 10 times the prevalence of detectable antibodies to *H. pylori* (33 vs. 3%). There was a significant inverse correlation between *H. pylori* antibodies and gestational age (ρ = −0.202, *P* = 0.05, *n* = 94).

C-reactive protein levels ranged from 0.2 to 109 mg/L. CRP levels were significantly lower in trimester 3 than in trimester 2 (*P* = 0.031) and CRP was inversely correlated with gestational age (ρ = −0.153, *P* = 0.047, *n* = 170). There was no association between CRP and cotinine. There was no significant change in WBC count during the three trimesters (Table 1).

While 47.6% of the women reported they were smokers, serum cotinine was detected in 50.4% of the mothers. The levels ranged from 0 to 440 ng/mL among women who were self-reported smokers. Cotinine was detected in two women who were self-reported non-smokers.

The levels of cortisol rose by trimester; levels were highest in the third trimester (Table 1; *P* < 0.001).

### Effects of risk factors on inflammatory markers

**Figure 1:**
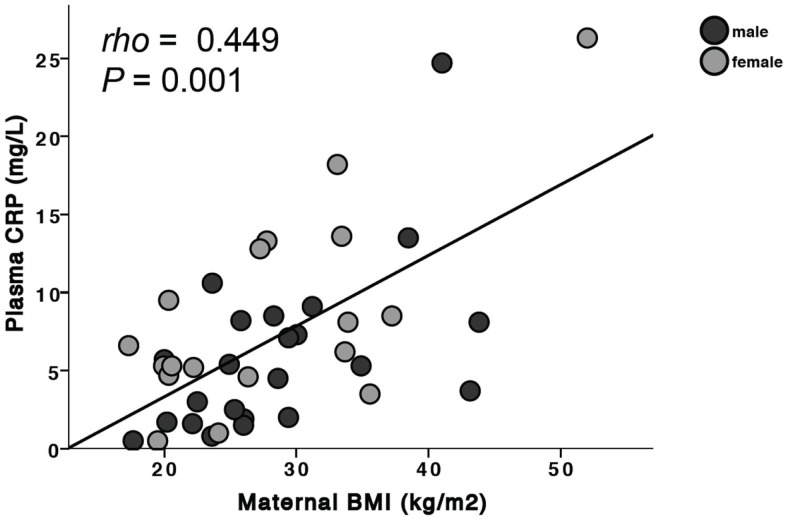
**Maternal BMI is positively associated with plasma CRP**. When separated by fetal sex, this was significant in mothers carrying a male fetus (males: ρ = 0.55, *P* = 0.007) but did not reach statistical significance in mothers carrying a female fetus (females: ρ = 0.442, *P* = 0.07).

Both WBC (ρ = 0.283, *P* = 0.003, *n* = 108) and IgG specific for *H. pylori* (ρ = 0.216, *P* = 0.032, *n* = 99) were associated with cotinine levels. In pregnancies carrying a male fetus, there was no association between IgG, IgA, or IgM levels and maternal BMI. There were, however, significant correlations in women carrying a female baby between maternal BMI and both IgG (ρ = 0.59, *P* = 0.01, *n* = 18) and IgA (ρ = 0.58, *P* = 0.012, *n* = 18). There was no association between any of the risk factors and the sex of the fetus with IgG antibodies to *H. pylori*. For WBC, a significant positive association with cotinine was found in mothers carrying either a male (ρ = 0.32, *P* = 0.037, *n* = 44) or female fetus (ρ = 0.285, *P* = 0.05, *n* = 48). Overall, CRP levels were significantly correlated with maternal BMI (ρ = 0.449, *P* = 0.001, *n* = 56, Figure 1); this was significant in mothers carrying male fetuses (ρ = 0.55, *P* = 0.007, *n* = 23) but not in mothers carrying female fetuses (ρ = 0.442, *P* = 0.07, *n* = 18). There was a negative correlation between IgG and cortisol levels (ρ = −0.211, *P* = 0.027, *n* = 110).

### Effects of risk factors on birth weight

**Figure 2:**
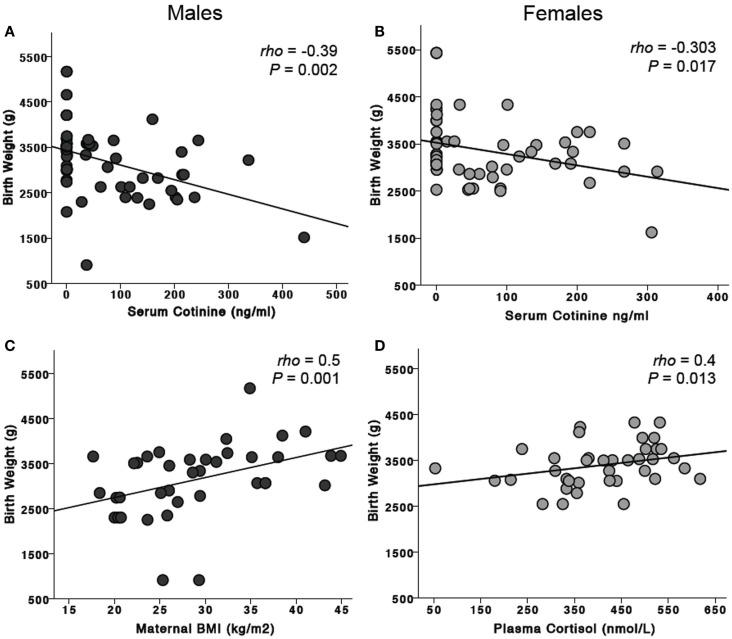
**Effects of risk factors on birth weight**. Maternal serum cotinine levels were negatively associated with birth weight in mothers carrying both male **(A)** and female **(B)** fetuses. Maternal BMI was significantly associated with birth weight for males **(C)** but not females. In contrast, maternal plasma cortisol was positively associated with birth weight for females **(D)** but not males.

The median birth weight for female infants was 3127.5 g (range 1620–5430 g) and that for male infants 3090 g (range 910–5170 g). Birth weight was significantly correlated with gestational age at delivery (ρ = 0.61, *P* < 0.001, *n* = 105). For all infants, maternal serum cotinine levels were negatively correlated with birth weight (ρ = −0.37, *P* < 0.001, *n* = 129, Figures 2A,B): males (ρ = −0.39, *P* = 0.002, *n* = 60); females (ρ = −0.303, *P* = 0.017, *n* = 62). For females, birth weight was positively correlated with maternal cortisol levels (ρ = 0.40, *P* = 0.013, *n* = 38, Figure 2D). This was not observed for males. Maternal BMI was significantly correlated with birth weight for all infants (ρ = 0.32, *P* = 0.005, *n* = 78). When assessed by sex of the infant, this was significant only for males (ρ = 0.5, *P* = 0.001, *n* = 38, Figure 2C).

### Effects of risk factors on gestational age at birth

**Figure 3:**
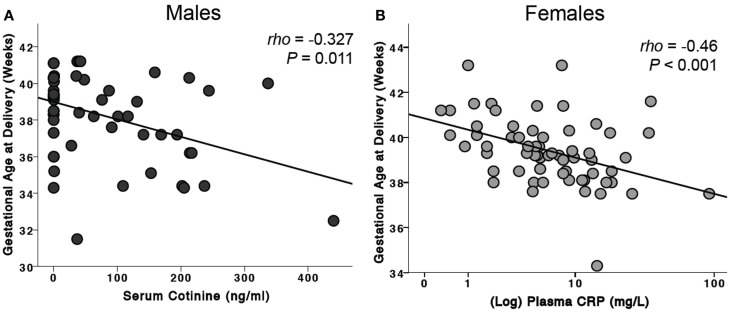
**Effects of risk factors on gestational age at birth**. **(A)** For male infants, serum cotinine was negatively associated with gestational age at delivery. This association was not seen in female infants. **(B)** Maternal plasma CRP levels were negatively associated with gestational age of delivery of female infants. This observation was not found in males.

For the combined male and female infants, maternal serum cotinine levels were negatively associated with gestational age at delivery (ρ = −0.199, *P* = 0.023, *n* = 129). When analyzed by sex of the fetus, this was significant only for males (ρ = −0.327, *P* = 0.011, *n* = 60, Figure 3A). CRP was negatively associated with gestational age only for female infants (ρ = −0.46, *P* < 0.001, *n* = 64, Figure 3B).

## Discussion

Our findings are discussed in relation to the hypothesis that Indigenous women have more powerful pro-inflammatory responses and that these might be enhanced by risk factors for SIDS, particularly smoking and high BMI. We chose our markers of inflammation based on results of a study of Indigenous Australians in a remote community (12) where for a cohort of 133 women, univariate analyses found CRP levels to be correlated with total IgG, IgA, and IgM, IgG antibodies to cytomegalovirus (CMV) and *H. pylori*, BMI, and cigarette smoking. Within this current study, we were unable to screen for IgG to CMV due to financial constraints. We assessed exposure to cigarette smoke by plasma cotinine, not just self-reported smoking in an effort to understand exposure to tobacco products in the home.

### Is there evidence of increased inflammatory responses among Indigenous women in this cohort?

Normal levels of CRP are considered to be <1 mg/L (13). The current study found that women in the first and second trimesters had median CRP levels slightly above 7 mg/L and these fell to 5.3 mg/L in the third trimester. This is similar to levels reported in non-pregnant Indigenous women of a similar age (12). A study of CRP in American women found that at 26 weeks gestation, median CRP was 4.8 mg/L; however, there were differences associated with ethnic group. Black mothers had higher values (7.68 mg/L) than white mothers (2.59 mg/L) even after data were controlled for smoking and maternal weight (11). In contrast to our findings, they reported increasing CRP levels with increased gestational age but showed no association between CRP and smoking (11). Both McDonald et al. and Picklesimer et al. reported a positive correlation between BMI and CRP levels (11, 12), however non-pregnant Indigenous women had a lower median BMI (23.7 kg/m^2^) than the pregnant women in our cohort (12).

Median IgG levels for women in our study (Table 1) were nearly half those reported for women in the remote community, 20.2 g/L (19.2–21.2). This might reflect a lower infection load compared with the women in the remote community, 68% of whom had evidence of IgG to *H. pylori* compared with a prevalence of 33% in our study. Total median IgA was also approximately three times higher in the remote community, 4.35 g/L (4.09–4.63) (12). Interestingly, there is a positive correlation between *H. pylori*-specific IgG and cotinine levels in our cohort. This association has been described previously (19) and indicates that exposure to cigarette smoke may contribute to the persistence of *H. pylori* infection. These data suggest that while the women in our cohort have similar levels of CRP compared to non-pregnant Indigenous women (12), this is likely to be related to increased obesity rather than a higher infection rate.

### Are inflammatory markers affected by risk factors associated with poor pregnancy outcome or sudden death in infancy?

Differences in inflammatory responses have been associated with the sex of the individual. In this study, we found that the sex of the fetus influences the levels of the mother’s inflammatory markers – total IgG levels, WBC, and CRP. Maternal BMI affected both IgG and IgA levels if the mother carried a female fetus. WBC levels were correlated with exposure to cigarette smoke for both male and female fetuses but were more significant for males. For CRP levels, there was a correlation with BMI; this was significant for women carrying a male fetus and only marginally significant for women carrying a female fetus. These results reflect those we have previously observed *in vitro*; there are significant effects of sex on inflammatory responses, possibly associated with testosterone levels (9) and these interactions require further investigation.

### Does inflammation affect birth weight or gestational age of male and female infants?

For all infants, maternal serum cotinine levels were negatively correlated with birth weight. For females, birth weight was correlated with maternal cortisol levels. BMI of the mother was significantly correlated with birth weight for combined male and female infants, but this was significant only in mothers carrying male fetuses.

For gestational age, there were negative correlations with cotinine levels, but these were significant only for males. There was a negative correlation with CRP levels but only in women carrying female fetuses.

The results indicate that male fetuses are more likely to be affected by risk factors that affect inflammatory responses that are associated with poor pregnancy outcome and sudden death in infancy – exposure to cigarette smoke and high maternal BMI. It is therefore not surprising that male infants are at greater risk of spontaneous preterm delivery (20–22), stillbirth (23, 24), and SIDS (25, 26).

## Conclusion

There is evidence for higher levels of inflammation in pregnant Indigenous Australian women. This might reflect the high levels of obesity or the high levels of both acute and chronic infections in this population. Levels of CRP were negatively associated with gestational age in female infants suggesting that mothers with chronic inflammation who are carrying a female fetus may be more likely to deliver their babies early.

This study also highlights the adverse effects of exposure to cigarette smoke on birth weight in both male and female infants, which has been well documented. Importantly, we have also shown that males appear to be more affected by exposure to cigarette smoking than females, as cotinine is negatively associated with gestational age at delivery. Being born preterm and being male puts these infants at a significant disadvantage in terms of their neonatal outcomes and their risk of death in infancy.

## Author Contributions

KP, KR, EL, and CB all made substantial contributions to the conception, design, analyses, and interpretation of the work. They assisted in preparing the article, critically assessed the final version, and agree to be accountable for the accuracy and integrity of the work. SH and CB made contributions to the analyses and interpretation of cotinine and the immunoglobulin studies undertaken. LW recruited all participants within the Indigenous pregnancy cohort and made substantial contributions to the study design and assisted with editorial aspects of the paper. RS made substantial contributions to the conception, design, analyses and interpretation of the work, and assisted with the final editorial aspects of the paper.

## Conflict of Interest Statement

The authors declare that the research was conducted in the absence of any commercial or financial relationships that could be construed as a potential conflict of interest.
